# Authors’ Response to Peer Reviews of “The Impact of SARS-CoV-2 Lineages (Variants) and COVID-19 Vaccination on the COVID-19 Epidemic in South Africa: Regression Study”

**DOI:** 10.2196/46944

**Published:** 2023-07-03

**Authors:** Thabo Mabuka, Natalie Naidoo, Nesisa Ncube, Thabo Yiga, Michael Ross, Kuzivakwashe Kurehwa, Mothabisi Nare Nyathi, Andrea Silaji, Tinashe Ndemera, Tlaleng Lemeke, Ridwan Taiwo, Willie Macharia, Mthokozisi Sithole

**Affiliations:** 1 The Afrikan Research Initiative Motloung South Africa

**Keywords:** COVID-19, infection, pandemic, vaccine, vaccination, epidemiology, transmissibility, health care, hospital admission, COVID-19 variants, SARS-CoV-2

*This is authors’ response to peer-review reports for “The Impact of SARS-CoV-2 Lineages (Variants) and COVID-19 Vaccination on the COVID-19 Epidemic in South Africa: Regression Study.”*

## Round 1 Review

### Reviewer AA [1]

The manuscript [2] is well written, and the subject addressed in this manuscript is worth investigating; however, the manuscript partly failed to present a clear picture of its analytical methodology and presentation of results.

Response: The authors have revised the methodology and presentation of the results.

The following are some minor concerns for consideration. I suggest that the authors (a) extend the study to include the recent Omicron variant.

Response: The authors have extended the study to include the Omicron variant.

The following are some minor concerns for consideration. I suggest that the authors (b) present results with complete models.

Response: The authors have presented results with complete model methodologies.

The following are some minor concerns for consideration. I suggest that the authors (c) avoid excessive references (~71).

Response: The authors have tried to reduce the references in the manuscript to critical references.

### Reviewer BQ [3]

#### General Comments

This manuscript is well written, comprehensive, and filled with detail. This is both a strength and a possible weakness. The strength is that the data included have been analyzed in depth, and one can be fairly certain that the results obtained are likely to be accurate. On the other hand, depending on the audience, some readers may struggle to engage with the data appropriately; the dissemination of data and reporting has not been formatted and simplified in a manner that improves readability without compromising on accuracy.

Response: The authors have reworded most of the sections, particularly the Results and Discussion to make the manuscript more reader friendly.

The use of scientific notation for P values to the 11th power, use of 3 or 4 decimal places for proportions, etc, and extensive reporting of findings instead of picking a few of the most relevant findings with reference to the table for other findings are a few examples of this.

Response: The presentation of P values in the manuscript has been reformatted as required.

#### Specific Comments

##### Major Comments

1. I have not seen whether time was included as a potential confounder/covariate in any of the regression models that were conducted. Increasing immunity, the initiation of vaccination campaigns halfway through the third wave, and movement restrictions have not been discussed adequately.

Response: The authors have included nonpharmaceutical interventions and COVID-19 vaccination as cofounders in the study analysis.

2. Please provide brief details on how data used to assess movement restriction were obtained and analyzed.

Response: Data on community movement was obtained from the Google Community Mobility reports. The regression of movement and the daily COVID-19 effective contact rate was then conducted through a literature review of earlier work done by the authors on this.

3. Please comment on the appropriateness of using means and standard deviations for the description of the majority of some of these data, which may or may not have been normally distributed.

Response: The authors have addressed this key question in the manuscript. For comparative inferential statistical analysis of continuous variables using the magnitude of the mean and variance, the distribution of the continuous variable must be the same in the periods being compared.

4. Please provide ethical considerations in the manuscript for the data and analysis, whether approval was required or not, and justify.

Response: Information used in this study was from public sources with creative commons licenses. The authors ensured reference data sources and acknowledged relevant institutions. The data used was blinded regarding patient personal information.

##### Minor Comments

1. “While, there is global consensus on the health risk posed by COVID-19, ground-breaking vaccine developments, and a great drive towards the vaccination of the world population against COVID-19.”

This sentence is fragmented. Please revise.

Response: The sentence was revised to “There is global consensus on the health risk posed by COVID-19, ground-breaking vaccine developments, and a great drive towards the vaccination of the world population against COVID-19. However challenges still persist in controlling the Global COVID-19 transmission and severity.”

2. “emergent.” Possible typo error, consider using “emergence.”

Response: Typo corrected.

3. National Coronavirus Command Council: A one-liner describing the National Coronavirus Command Council would be beneficial to the reader.

Response: The authors felt this would be unnecessary considering the word limit. The relevant reference has been added for the reader interested in looking for more information. The background has relatively low relevance to the study.

4. “Beta SARS-CoV-2 lineage required a half Maximal inhibitory concentration (IC50) 6 to 200 fold higher than the lineages identified in the first wave.” What reagent/antibody/method is used to test the IC 50 cited here?

Response: The authors wish to guide you to the following paper for more information. This background has relatively low relevance to the study, particularly the information on the reagent used. Cele S, Gazy I, Jackson L, Hwa SH, Tegally H, Lustig G, et al. Escape of SARS-CoV-2 501Y.V2 from neutralization by convalescent plasma. *Nature*. 2021;593(7857):142-146. doi:10.1038/s41586-021-03471-w.

5. “estimated that it was 1.29 (95%CI: 1.9601.58)).” Unsure what the confidence interval is there. Please review.

Response: This error has been corrected.

6. “period) showed significant difference at 95 % confidence interval between the respective COVID-19 epidemic periods with p-values of 1.82×10-11 and 5.87×10-05 respectively.”

The author team can check submission guidelines, and the editor can confirm, but I believe that P values <.001 should be stated as such.

Response: The presentation of P values in the manuscript has been reformatted as required.

7. Table entries with variable names that have underscores and labeling could be cleaned up to improve readability.

Response: The use of the underscore was left unchanged as the authors feel this is the best method of referencing the epidemic waves in multiple variables of the study. This is also described in the methodology for the reader to understand their meaning (underscore and number).

8. As noted above, the use of 3 or 4 decimal places and exponential notation of extremely small P values reduces the clarity and readability. Consider reviewing.

Response: The presentation of P values in the manuscript has been reformatted as required.

### Reviewer Anonymous [4]

#### Specific Comments

The article seems good to me but too complex and difficult to follow, it should be “lightened.”

Response: The authors have restructured the paper for easier readability.

##### Major Comments

When talking about COVID-19 and its variants, some important points should be clarified that inform and prepare the reader well to deal with the specifics. Therefore, to make this paper more complete and interesting for the readers of this important journal, the authors should expand a bit of the discussion on cytokines. On this subject, three important articles have recently been reported. Below I list these interesting articles that should be studied, incorporated into the meaning, and reported briefly in the discussion and in the list of references.

- Conti P, Caraffa A, Tetè G, Gallenga CE, Ross R, Kritas SK, et al. Mast cells activated by SARS-CoV-2 release histamine which increases IL-1 levels causing cytokine storm and inflammatory reaction in COVID-19. *J Biol Regul Homeost Agents*. 2020;34(5):1629-1632. PMID:32945158 doi:10.23812/20-2EDIT
- Ronconi G, Teté G, Kritas SK, Gallenga CE, Caraffa A, Ross R, et al. SARS-CoV-2, which induces COVID-19, causes kawasaki-like disease in children: role of pro-inflammatory and anti-inflammatory cytokines. *J Biol Regul Homeost Agents*. 2020;34(3):767-773. PMID:32476380 doi:10.23812/EDITORIAL-RONCONI-E-59
- Conti P, Caraffa A, Gallenga CE, Ross R, Kritas SK, Frydas I, et al. Coronavirus-19 (SARS-CoV-2) induces acute severe lung inflammation via IL-1 causing cytokine storm in COVID-19: a promising inhibitory strategy. *J Biol Regul Homeost Agents*. 2020 Nov-Dec;34(6):1971-1975. PMID:33016027 doi:10.23812/20-1-E

Response: The authors found the suggested papers interesting. The following paper “Mast Cells Activated by SARS-CoV-2 Release Histamine Which Increases IL-1 Levels Causing Cytokine Storm and Inflammatory Reaction in COVID-19” was included in the paper as a reference; however, the authors, due to the word limit, did not expand on this topic. Though interesting, it has low relevance to the study.

##### Minor Comments

Some legends should be expanded.

I believe these suggestions are important for improving this paper. Without these corrections, the paper cannot be published. So I recommend minor revision.

Response: Legends in the paper were expanded.

### Reviewer Anonymous [5]

#### Specific Comments

##### Major Comments

1. Throughout the manuscript, the notation of numbers is not consistent. For example, in the middle of the second paragraph in section 1, Introduction, “The genome of SARS-CoV-2 is a single positive-stranded RNA approximately 29 903 bases (nucleotides) pairs in length 9 [6-9].” It looks like a space between numbers indicates a digit of a thousand, and a comma is omitted. However, in the middle of the paragraph in section 2.2.1., “Table 2 shows that the mean COVID-19 daily tests in the first, second and third South African COVID-19 epidemic wave period were 20 575±14 062, 31 046±14 115 and 46 822±18 460 respectively.” A space between numbers indicates a decimal point, not a comma.

Response: The authors have corrected this error. A space between numbers in the manuscript represents a digit of a thousand.

2. Sections 2 and 3 are extremely difficult to read because they are too lengthy, although subsections indicate each statistical analysis that was performed. I believe that the authors do not need to provide outputs copied from SPSS directly. Are all columns in each table meaningful? Should readers know both standard deviation and variance for each statistic, for example? I strongly suggest that the authors get rid of unnecessary columns in each table and move unnecessary tables from sections 2 and 3 to the appendix.

Response: The authors have reduced the columns in the tables and moved some of the tables to the appendix. The authors have also rewritten these sections for easier readability.

3. I believe that the P values in the manuscript do not need to be specific. For example, Table 3 displays Pearson and Spearman correlation coefficients and P values. Many people may not understand what 9.94E-79 means. It can be simplified to “<0.001” or 0.

Response: The presentation of P values in the manuscript has been reformatted as required.

##### Minor Comments

4. The font style and size are not consistent throughout the manuscript.

Response: The font and style have been made consistent throughout the manuscript.

## Round 2 Review

### Reviewer BQ [3]

The manuscript has been improved based on previous reviewer comments but is still unnecessarily too long, dense, and bloated. I believe that the adage “simpler is better” would have suited the objectives of this paper well. The average reader may find it difficult to read to the end, and some readers may have difficulty fully engaging with the content as a result. Five pages on the virology of SARS-CoV-2 as an introduction is likely unnecessary for a manuscript whose data focus on the epidemiology and statistics of COVID-19 rather than its virology.

Response: The authors agree with this review note and have cut down the Introduction (to 2.25 pages) to focus on the background of detected SARS-CoV-2 and COVID-19 vaccination in South Africa to prepare the reader for the study objectives.

There are many statistical tests conducted here; however, the authors do not appear to have performed any adjustments for the multiple tests conducted. The familywise error rate is bound to be higher than 0.05, so some of your conclusions based on the statistical probability may be inaccurate.

Response: Each descriptive and inferential statistical analysis conducted/applied on the analysis data sets and conclusions drawn from each inference were done independently as per the objective of the statistical analysis method. However, type 1 error are noted and covered in the limitations stated in the manuscript under Data Handling and Limitations.

Finally, there are some statements that have been made based on the Discussion and Conclusion sections that I do not believe are adequately supported by the data presented, and these may need to be reconsidered/softened. Please see specific comments below.

Response: Thank you for this review. The authors agree with your statements below.

1. Methods: Many hypothesis tests are conducted in this paper. Was adjustment for multiple testing performed? Otherwise, the possibility of making type 1 errors is quite high. This should either be reviewed or listed as a key limitation.

Response: The limitations of the manuscript have been listed under Data Handling and Limitations. Statistical tests were applied independently; however, the potential for type I or II errors has been noted.

2. South Africa community mobility data: How is movement in these data measured? Kilometers? Significant movement out of the house? The number of people in an area? Please describe.

Response: The Google Mobility reports are created with aggregated data from users who have turned on their Location History in their Google accounts. The baseline in these reports is the median values of movement in the respective locations from January 3 to February 6, 2020. This movement unit is the percentage from baseline (number of people in that location per time relative to the number observed at baseline).

3. “The mean daily positive COVID-19 tests in South Africa’s first and second COVID-19 epidemic wave had no statistically significant difference.”

Please report the data and P values or reference the table where these data can be found.

Response: P value added to this statement.

4. Please insert a legend for the figures (eg, Figures 7 and 8).

Response: Legends inserted for the figures.

5. Table 1: The maximum COVID-19 hospitalized intensive care unit percentage of 7 and 814.1 is unclear.

Response: This statement was removed in the writing of the Discussion section.

6. Discussion: “The values of the Pearson and Spearman Correlation Coefficients obtained between the daily COVID-19 tests and cases in this study indicated a strong positive correlation between daily COVID-19 tests and cases with more than 95 % confidence in the four COVID-19 epidemic waves in South Africa.”

Please review this interpretation of your correlation significance and 95% confidence intervals. It is technically incorrect to say that “there is more than 95% confidence.”

As a suggestion, you may leave the 95% confidence part out altogether and just say that testing was significantly related to case incidence in the 4 COVID-19 waves.

Consider also reviewing the American Statistical Association papers on P values and moving toward more conservative reliance on statistical significance overall (Wasserstein RL, Schirm AL, Lazar NA. Moving to a world beyond “*P*<0.05”. *Am Statistician*. 2019;73(sup1):1-19. doi:10.1080/00031305.2019.1583913).

Response: The statement was changed to “The values of the Pearson correlation coefficients obtained between the daily COVID-19 tests and cases in this study indicate a strong positive association between daily COVID-19 tests and cases in the five COVID-19 epidemic waves in South Africa,” and the “95% confidence” was removed.

7. These data, as presented, do not allow you to make this conclusion as you have not made a relationship of causality, but rather have demonstrated an association, as you rightly say in the following lines. Please revise to describe this as a significant association rather than a causal relationship.

Response: Instead of relationship, the word “association” was used to avoid an interpretation of causality instead of correlations.

8. “To understand the causality of relationships between two or more variables, statistical theory must be applied.” Text like this is unnecessary and contributes to the bloating of your manuscript. Consider removing.

Response: This statement was removed in the rewriting of the Discussion section.

9. “Daily COVID-19 tests in South Africa were observed to be normally distributed while the daily COVID-19 cases were positively skewed with a lognormal distribution (Galton distribution).”

I do not recall the data distributions being assessed or described in the Results, so it is surprising that they are now included in the Discussion. Consider including or revising the need to discuss the data distributions (a similar comment applies to the following paragraph).

Response: The discussion of variable normal distributions was removed from the manuscript.

10. I have reservations about the use of the word “confounder” in this discussion. While the movement is most likely a potential contributing factor in the detection rate of COVID-19, this was not analyzed or demonstrated using appropriate statistical methods such as multiple regression or interaction tests.

Showing that there was a correlation between population movement and COVID-19 detection does not automatically demonstrate that movement is a significant confounder. The messaging may have to be altered to suggest a possible confounding effect, or alternatively, this would need to be demonstrated by conducting appropriate data analysis.

Response: The words “possible” and “association” were used since there were not enough multivariable statistical methods applied in the manuscript to avoid conclusive statements.

11. “The values of the Spearman Correlation Coefficients obtained between the daily cumulative COVID-19 vaccinated people and change in daily COVID-19 cases in the half period of the third and fourth COVID-19 epidemic wave in this study indicated a low correlation between the daily cumulative COVID-19 vaccinated people and change in daily COVID-19 cases with this correlation statistically insignificant.”

This statement should be reconsidered. If vaccination does indeed have a significant effect on daily infection rates, there is bound to be a lag between exposure and effect, and this would need to be demonstrated in a robust time series analysis. Correlating the vaccination rate with the COVID-19 case rate without adjustment for time periods would not adequately demonstrate the effect of vaccination if such an effect existed. This is particularly important because the statement “These results suggest that COVID-19 vaccines administered in South Africa had no significant effect on the transmission of COVID-19” would be a controversial conclusion to come to without solid evidence to support this statement that may be seen as inflammatory in the politically charged topic of vaccines and vaccine hesitancy in South Africa.

Response: This statement was removed from the Discussion. The authors agree that there is not enough evidence generated in the results of the manuscript to make this conclusion.

12. “This result can be explained by the percentage of the population per age group who had received at least one dose of the COVID-19 vaccine by the end of the fourth COVID-19 epidemic wave.”

This statement appears to contradict your earlier statement that vaccines did not appear to have an impact on COVID-19 transmission in South Africa. Please review and reconcile. Also, natural immunity and potentially reduced virulence of the Omicron variant are important factors to consider in the reduced mortality in the fourth wave.

Response: The statements on the impact of the COVID-19 vaccine on transmissibility were retracted in the manuscript due to insufficient evidence from the available data. Including this conclusive analysis will require data that captures COVID-19 daily cases and their vaccination status.

13. “showed statistical significant indifferences at 95 % confidence.” Unusual wording and terminology such as indifference at 95% confidence. Please revise.

Response: The wording has been revised.

14. “While COVID-19 vaccines administered in South Africa had no significant effect on the transmission of COVID-19 within the South African population.”

Again, this statement is not supported by the data provided and should be reviewed and reconsidered.

Response: The statements on the impact of the COVID-19 vaccine on transmissibility were retracted in the manuscript due to insufficient evidence from the available data. Including this conclusive analysis will require data that captures COVID-19 daily cases and their vaccination status.

15. Table A. 1: Consider formatting these large sums of square and mean square values including thousand separators for readability.

Response: Commas to separate thousands were included in the formatting of all numbers in the manuscript.

### Reviewer Anonymous [5]

#### General Comments

The authors have tried to improve the quality of the manuscript. However, the manuscript still needs substantial improvement. Please see my comments.

Response: Thank you for this review. The authors agree with your statements below.

#### Specific Comments

##### Major Comments

1. This issue has not been resolved. The authors said that the space between numbers indicates a digit of a thousand. However, according to JMIR house style and editorial guidelines, numbers greater than 999 have a comma to separate thousands, millions, etc. Please see [10] and update the style of numbers throughout the manuscript.

Response: Commas to separate thousands were included in the formatting of all numbers in the manuscript.

2. The authors have reduced unnecessary columns. However, the JMIR production team suggests no more than 5 tables per manuscript. There are still unnecessary tables in the manuscript, that do not provide meaningful information and are just the same outputs of SPSS. What is the purpose of including so many tables without interpretation? Should Table 1 really be placed in the main manuscript? Why? Please see [11].

Response: The authors have moved unnecessary tables and figures to the appendix.

3. The authors have updated the representation of P values according to the suggestion of the editorial director [12].

Response: No updates required.

4. The font style is still not consistent throughout the manuscript. Please revise the font style.

Response: The font style has been revised and made consistent throughout the manuscript.

5. The Introduction in the manuscript is too long. I would suggest reducing the Introduction in the manuscript.

Response: The authors agree. The Introduction in the manuscript was reduced.

6. There are 13 equations in the manuscript. I believe that the authors can reduce the number of equations in the manuscript by combining similar equations. Listing all equations is unnecessary. Also, reference numbers for equations could be a number in the parenthesis such as (1) instead of Equation 1.

Response: The authors have removed unnecessary equations in the manuscript.

7. Detailed information about the paired test (what pairs to what) will be placed in the footnote in the corresponding table or figure.

Response: This was removed from the captions of the tables and described in the methodology.

8. Why do the authors think that the following text or Table 3 is needed in the manuscript?

“Table 3 shows that the Pearson (Spearman) Correlation Coefficients between COVID-19 daily tests (Independent Variable) and cases (Dependent Variable) in the first, second, third and fourth COVID-19 epidemic wave in South Africa were 0.910 (0.955), 0.877 (0.751), 0.893 (0.847) and 0.854 (0.812) respectively.”

This text and Table 3 are the same information.

Response: Table 3 was moved to the appendix and the text was used instead for the Results section.

9. What is the reason to provide Pearson correlation and Spearman rho together? Do the authors want to show a linear relationship or an ordinal relationship?

Response: The authors used throughout the Spearman rho correlation coefficient and left the Pearson correlation for normally distributed variables.

##### Minor Comments

10. The footnotes in Tables 3 and 4 are redundant. Where are the superscripts a, b, or c in the tables?

Response: Footnotes in Tables 3 and 4 were removed, and the tables were moved to the appendix.

11. There is an inconsistent number of digits in all tables in the manuscript.

Response: The authors agree and have resolved all formatting of numbers according to JMIR guidelines.

12. From Tables 1 to 16, why do the authors think that the minimum and maximum provide meaningful information in Table 2?

Response: The minimum and maximum provide the lowest and highest values observed in the epidemic wave period, which corresponds to the start/end and the peak of the epidemic wave period.

13. Please use “95% confidence interval” instead of “95 % confidence interval.”

Response: “95% confidence interval” was used instead, and all percentage values were formatted accordingly.

## Round 3 Review

### Reviewer BQ [3]

#### Comments

1. Table 8: Consider having the case-fatality age risk ratio value for the reference group as “Ref” for reference. It may be confusing to have a risk ratio for the reference category.

Response: Updated the caption of Table 8 and the values of the case-fatality age risk ratio reference group to make the case-fatality age risk ratio reference clearer.

2. Table 9: Case-fatality rate is abbreviated as “CRF” at times (and in subsequent text) and as “CFR” at times.

Response: The abbreviation of case-fatality rate in Table 9 and Table A12 was corrected to “CFR.” The in-text reference to the case-fatality rate abbreviation was checked to ensure they are all abbreviated as “CFR.”

### Reviewer Anonymous [5]

#### Major Comments

1. In “Covariance and Regression of South African Epidemiological Data,” the authors stated that the 2-tailed Pearson correlation above 0.850 with P<.001 was considered as having a high degree of linearity. Pearson correlation coefficient has a value between –1 and 1. A negative value (eg, –0.850) could also be considered as a strong negative relationship between two variables. Was a negative relationship included in the determination of linearity?

Response: Thank you for this comment. The authors agree with the reviewer. Indeed, a value of less than –0.850 implies a strong negative relationship/association between two variables. The authors did conduct their analysis in this manner; however, unfortunately, the wording was omitted in the methodology. The Methods section Covariance and Regression of South African Epidemiological Data has been updated to include “or below -0.850.”

2. In “Normalisation and Paired T-tests on South African Epidemiological Data,” the authors considered only 7 pairs among 5 periods. Normalized parameter 2 and 4, normalized parameter 2 and 5, and normalized parameter 3 and 5 were not included in pairing. Was there a specific reason to exclude these three pairs in the paired *t* test?

Response: The authors initially did consider having all possible test pairings; however, it would have complicated the analysis. We, therefore, chose two analysis groupings in terms of test pairing. The first one was comparing all COVID-19 epidemic waves to the first COVID-19 epidemic wave (pair 1 to pair 4). This would help us understand the impact of the evolution of SARS-CoV-2 (inclusive of other factors: nonpharmaceutical interventions, vaccination, etc) against the ancestral SARS-CoV-2 lineage (and initial conditions). The second analysis grouping was understanding the evolution per consecutive waves (pairs 5, 6, and 7). This would help us understand the impact of the evolution of SARS-CoV-2 (including changing conditions) between each consecutive wave. This simplified the analysis and allowed us valid inference between test pairings and an overview based on the two analysis test pairing groupings.

3. In the Discussion, the authors stated that the Pfizer-BioNTech (Comirnaty) and the Johnson & Johnson/Janssen COVID-19 vaccines have shown high efficacy against severe COVID-19 at 85% and 88.9%, respectively. However, two terms, vaccine efficacy and effectiveness, are used in different settings. According to [13], Pfizer demonstrated their COVID-19 vaccine efficacy based on randomized controlled trials. However, Johnson & Johnson did not show their COVID-19 vaccine efficacy according to [14]. Instead, Johnson & Johnson demonstrated their COVID-19 vaccine effectiveness based on observational studies, which is in a real-world setting. Could you please clarify this? (Please see [15].)

Response: Thank you for this comment, and it touches on an important discourse regarding the implications of using different methodologies to infer efficacy, with of course, randomized clinical trials being the standard. Certainly, the authors accept the reviewer’s point; for the Discussion, the authors wanted to highlight these studies for reference in terms of the efficacy against severe COVID-19. Unfortunately, there are, of course, limitations in the inference of efficacy, as it does depend on the methodology of those studies. The authors in the manuscript used the reference to allow the reader to understand the current work regarding the association, which is highlighted by the manuscript (increasing vaccination, decreasing hospitalization). In light of the reviewer's point, we have updated reference [14] to Sadoff J, Gray G, Vandebosch A, Cárdenas V, Shukarev G, Grinsztejn B, et al. Safety and efficacy of single-dose Ad26.COV2.S vaccine against COVID-19. *N Engl J Med*. 2021;384:2187-2201. PMID:33882225 doi:10.1056/NEJMoa2101544.

#### Minor Comments

4. The authors did not explain what the special characters after SARS-CoV-2 variants mean (eg, BA.4# or BA.2.75***). Could you please provide details on what the special characters after SARS-CoV-2 variants indicate?

Response: The naming of these lineages with special characters “#” or “*” appeared due to an error in rendering our document. We have updated to remove these from the naming of the lineages.

5. The authors used unnecessary abbreviations throughout the manuscript. Could you please review the manuscript and remove some unnecessary abbreviations that are not used in a section of the manuscript?

Response: The authors reviewed the abbreviations used in the manuscript and removed unnecessary abbreviations.

## Round 4 Review

### Reviewer Anonymous [5]

#### Specific Comments

##### Major Comments

1. It is difficult to understand what Tables 2 and 3 show. Table 3 provides the mean difference between two daily positive COVID-19 tests in a percentage. If we look at the paired differences mean of pair 5 (daily positive COVID-19 test 2 – daily positive COVID-19 test 3), the difference is –1.20. However, the mean of the daily positive COVID-19 test 2 is 11.5 and the mean of the daily positive COVID-19 test 3 is 13.3 in Table 2. Could you please clarify what you compare between the two groups? How do we understand Tables 2 and 3 together? The same comment will be applied to Tables 4 and 5.

Response: Table 2 shows the descriptive statistics for the COVID-19 active cases and daily positive COVID-19 tests (%, ie, what percentage of the total COVID-19 tests were positive) for each epidemic wave. The descriptive statistics include the number of valid observations (n), minimum, maximum, mean, and standard deviation (std deviation).

While, Table 3 shows the paired sample *t* test results between test pairing (ie, between epidemic waves), showing the paired differences of the mean and standard deviation, the student *t* test value, degrees of freedom (*df*), and the P value.

Now discussing the pairings you are comparing, pair 5 in Table 3 is the comparison between the daily positive COVID-19 tests in the COVID-19 epidemics 2 and 3. The paired mean difference was –1.20; however, the actual mean difference (13.3 – 11.5) is 1.80 as you have stated. The discrepancy between Tables 2 and 3 is due to the degrees of freedom (*df*) in Table 3 and observations (n) in Table 2. Test pairing was done based on the epidemic day; therefore, the epidemic wave with the lowest observations will always be the *df* of the *t* test. We have to compare like with like; due to this, some of the observations in Table 2 are not included in the *t* test. This concept is the same for Tables 4 and 5.

##### Minor Comments

2. The notation of P values throughout the manuscript is inconsistent.

On page 5, “with Pearson correlations above 0.850 or below -0.850 with P<.001 considered as having a high degree of linearity.” On page 8, “The Spearman’s correlation coefficients and P-values between the daily cumulative COVID-19 vaccinated people and the daily COVID-19 cases in the first half period of the third, fourth and fifth COVID-19 epidemic wave in South Africa were 0.930 (95% CI 0.890-0.956), 0.842 (95% CI 0.713-0.916) and 0.811 (95% CI 0.673-0.895) respectively with P-values<.001. While the Spearman’s correlation coefficients and P-values between the daily cumulative COVID-19 vaccinated people and the change in daily COVID-19 cases were 0.031 (P=.79 95% CI -0.207-0.266), -0.014 (P=.93 95% CI -0.341-0.316) and -0.077 (P=.62 95% CI -0.374-0.233) respectively.” Could you please make an update on the notation?

Response: The authors have updated the notation of P values in the manuscript. The authors have followed the recommendations in [12].
